# Potential Utility of Novel Biomarkers in Active Surveillance of Low-Risk Prostate Cancer

**DOI:** 10.1155/2015/475920

**Published:** 2015-08-03

**Authors:** Jeong Hyun Kim, Sung Kyu Hong

**Affiliations:** ^1^Department of Urology, Kangwon National University School of Medicine, Chuncheon, Republic of Korea; ^2^Department of Urology, Seoul National University Bundang Hospital, Bundang-gu, Seongnam 463-707, Republic of Korea

## Abstract

Active surveillance (AS) is now an accepted management strategy for men with low-risk localized prostate cancer (PCa). However, detecting disease progression in a patient selected for AS remains a challenge. It is crucial to know what will serve as the best parameter to correctly identify tumors that progress to a more aggressive phenotype so as not to miss the window of curability. Several biomarkers are now being actively investigated as novel tools to improve PCa risk assessments. To date, several serum, urinary, and tissue biomarkers have shown promising prognostic value. %[−2]proPSA and PHI showed improved predictive value for an unfavorable biopsy conversion at annual surveillance biopsy in the AS program. PCA3 and TMPRSS2:ERG had additional independent predictive value for the prediction of PCa detection and progression, although PCA3 was limited in predicting aggressive cancer. Other tissue biomarkers also showed promising ability to predict disease progression. Although several of these novel biomarkers have an improved predictive accuracy that is better than classical parameters, there is still a need for further well-designed, large, multicenter, prospective trials to avoid common bias and clinical validation.

## 1. Introduction

Active surveillance (AS) is now an accepted management strategy for men with low-risk localized prostate cancer (PCa), as the majority of men with such cancers are unlikely to die of PCa [1–3]. Nevertheless, as low-risk cancer does not mean complete absence of risk, the large majority of men with low-risk, early-stage disease undergo aggressive intervention with radical prostatectomy (RP) and/or radiation therapy (RT), despite their attendant long-term side effects and cost [4–6]. Detecting disease progression in a patient selected for AS remains a continuing challenge. It is a crucial issue to determine what will serve as the best parameter to correctly identify tumors that progress to a more aggressive phenotype in order not to miss the window of curability. Approximately one-third of the patients will be reclassified as a higher risk for progression and will be offered treatment during AS [7–10]. In most cases that are reclassified as higher risk, the reclassification is due to upgrading at the time of a repeat biopsy [7–10]. This upgrading is largely not time dependent, suggesting that it is due to more accurate sampling rather than true biologic progression [11]. Some patients with apparently low-risk disease actually harbor unfavorable disease due to inaccuracies in the currently used repeat biopsy protocols [8]. Nevertheless, current AS criteria may be too strict, thereby excluding some patients in whom expectant management would be appropriate and safe [12].

Currently, serial PSA measurements, digital rectal examination (DRE), and repeat prostate biopsies are being used for risk stratification of men with early-stage PCa in most AS cohorts. Although these tools have some predictive value, a substantial fraction of men that are expected to have low-risk disease are found to have more aggressive disease at prostatectomy. The role of PSA and PSA kinetics still remains contentious. The AS program at Johns Hopkins does not use PSA changes as a trigger for curative intervention [10]. Although, in the Toronto series, a PSA DT < 2 years has been used to prompt treatment, this group currently does not use PSA kinetics alone as a trigger for treatment, but rather to trigger either rebiopsy or multiparametric MRI (MP-MRI) [13]. Furthermore, although morbidity is low [14], the discomfort, cost, and continued undersampling problem inherent in the prostate biopsy procedure advocate for the development of noninvasive tools capable of predicting disease progression more accurately and suitable for repeat measurements over time.

Accordingly, there is an unmet need for a noninvasive biomarker test that can provide a higher degree of specificity for detecting aggressive disease than the currently available clinical tools. Several biomarkers are now actively investigated as novel tools to improve patient selection and monitoring on AS for low-risk PCa.

## 2. Materials and Methods

### 2.1. Evidence Acquisition

We conducted a systematic review by the search of the PubMed database according to the Preferred Reporting Items for Systematic Reviews and Meta-Analysis statement guidelines (http://www.prisma-statement.org). Predefined search terms were used to identify articles published before August 5, 2014, for combinations of the following free search terms: “Biomarkers” and “Active Surveillance” or “Watchful Waiting” and “Risk Assessment” and “Prostatic Neoplasms.”

### 2.2. Search Results

The literature search identified 39 original articles that were included for review: 12 on %[−2]proPSA and Prostate Health Index (PHI), 10 on PCA3, 7 on TMPRSS2:ERG, 2 on genomic prostate score (GPS), 5 on the panel of four kallikrein markers, and 3 on cell cycle progression (CCP) score.

## 3. PSA Isoform and Its Derivatives

Free PSA (fPSA) includes the subforms benign prostatic hyperplasia-associated PSA (BPSA), inactive PSA (iPSA), and proPSA [15–17]. BPSA and iPSA are associated with benign tissue, but proPSA is associated with cancer [15–17]. It is possible to detect three truncated forms of proPSA in serum, [−2], [−4], and [−5, −7], with [−2]proPSA being the most stable form. A [−2]proPSA assay showed the clinically acceptable analytical performance with excellent precision and reproducibility and had negligible interference with other PSA isoforms [18]. Development of the [−2]proPSA immunoassay by Beckman Coulter opens a new field of study for detecting PCa.

Several studies [19–25] have suggested that, in men with a total PSA (tPSA) between 2.5 and 10 ng/mL, %[−2]proPSA (the percentage of [−2]proPSA to fPSA) and Prostate Health Index (PHI; ([−2]proPSA/fPSA) × (tPSA)^1/2^) provide significantly better clinical performance for predicting PCa than total PSA or %fPSA and may be related to PCa aggressiveness, with higher levels of these tests being in patients with Gleason score ≥ 7 (Table 1).

In an observational, prospective, multicenter European study (*n* = 646) [22], [−2]proPSA, %[−2]proPSA, and PHI significantly increased the accuracy of the base multivariate model by 6.4%, 5.6%, and 6.4%, respectively (*P* < 0.001). At a PHI cut-off of 27.6, a total of 100 biopsies (15.5%) could have been avoided. Moreover, %[−2]proPSA and PHI were significantly correlated with Gleason score (*ρ* = 0.245; *P* < 0.001 and *ρ* = 0.276; *P* < 0.001, resp.).

Interestingly, the same group also reported that %[−2]proPSA and PHI are more accurate than tPSA, fPSA, and %fPSA for predicting PCa in men with a family history of PCa (*n* = 158) from the PROMEtheuS cohort [23]. At a %[−2]proPSA threshold of 1.20 and a PHI threshold of 25.5, 24.8% and 17.2% of prostate biopsies could have been avoided, respectively. Moreover, [−2]proPSA, %[−2]proPSA, and PHI were directly correlated with cancer aggressiveness in patients with PCa in this study.

In a recently published large multicenter study (*n* = 1,362) [24], %[−2]proPSA and PHI had better clinical performance for predicting PCa compared with other PSA derivatives (area under the curve (AUC); PHI = 0.74, %[−2]proPSA = 0.72, [−2]proPSA = 0.63, %fPSA = 0.61, and tPSA = 0.56, resp.). Significantly higher median values of %[−2]proPSA and PHI were observed for patients with a Gleason score ≥ 7 (%[−2]proPSA = 2.68 and PHI = 60) compared with a Gleason score < 7 (%[−2]proPSA = 2.34 and PHI = 53) (*P* = 0.011 and *P* = 0.0018, resp.).

In a meta-analysis by Filella and Giménez [26], measurement of %[−2]proPSA and PHI showed improved accuracy for detecting PCa compared with that of PSA or %fPSA, as well as a good relationship with cancer aggressiveness, particularly in the group of patients with PSA of 2–10 ng/mL.

Moreover, [−2]proPSA-based parameters including PHI appear to provide improved predictive value for biopsy reclassification during AS follow-up. Makarov et al. [27] evaluated the potential association of serum and tissue proPSA levels for predicting patients who will develop an unfavorable biopsy conversion (Gleason ≥7 or ≥ 3 positive cores or > 50% of any core involvement) on annual surveillance examination, using serum and prostatic biopsy samples from 71 men in a Johns Hopkins AS program. They found that the ratio of [−2]proPSA to %fPSA in serum was significantly higher at diagnosis in men developing unfavorable repeat biopsy compared to the favorable repeat biopsy group (0.87 ± 0.44 versus 0.65 ± 0.36 pg/mL; *P* = 0.02). In their extended investigation to incorporate PHI in this same cohort, [−2]proPSA/%fPSA (*P* = 0.004) and phi (*P* = 0.003) were also significant predictors of unfavorable biopsy conversion in a Cox regression analysis [28]. According to this study, PHI and [−2]proPSA/%fPSA, combined with biopsy tissue DNA content, improved accuracy to about 70% to predict unfavorable biopsy conversion at the repeat biopsy among men enrolled in an AS program.

Tosoian et al. [29] (*n* = 167) also reported that baseline and longitudinal %fPSA, %[−2]proPSA, [−2]proPSA/%fPSA, and PHI measurements are significantly associated with biopsy reclassification, and %[−2]proPSA and PHI provided the greatest predictive accuracy for high-grade cancer during AS follow-up. Recently, Hirama et al. [30] evaluated the predictive impact of baseline [−2]proPSA and related indices on the pathological reclassification at 1 yr in 67 of 134 candidates for AS. Multivariate logistic regression analysis revealed baseline %[−2]proPSA and PHI (both *P* = 0.008) to be the only independent predictive factors for pathological upgrading 1 yr after beginning AS. However, that study was limited by a short follow-up period (1 yr), because reclassification at short follow-up period during AS might be mostly due to more accurate sampling rather than true biologic progression.

Although studies evaluating the potential role of %[−2]proPSA and PHI in an AS program are currently scarce, we found improved predictive value for an unfavorable biopsy conversion at annual surveillance biopsy in the AS program. Additional validation is warranted to determine whether clinically useful thresholds can be defined and to better characterize the role of %[−2]proPSA and PHI in conjunction with other markers in monitoring patients enrolled in AS in the future.

## 4. PCA3

Prostate cancer antigen 3 (PCA3) is a prostate-specific noncoding mRNA detectable in urine and greatly overexpressed in PCa compared with benign tissue [31–33]. Measuring PSA mRNA allows for the standardization of the number of PCA3 RNA copies by calculating the ratio of PCA3 to PSA (PCA3 score). Despite its cost, PCA3 outperformed PSA and %fPSA for early detection of PCa [34]. In a meta-analysis of the clinical utility of urinary PCA3 for diagnosing PCa [35], sensitivity was 54–82% and specificity was 66–89%, with AUC of 0.66–0.87. Several studies [36–38] have investigated the correlations between PCA3 score and PCa aggressiveness features, including tumor volume, Gleason score, pT stage, and percentage of positive biopsy cores.

Marks et al. [36] (*n* = 226) demonstrated the superiority of PCA3 over PSA by using a third-generation PCA3 assay (Gene Probe Progensa) (AUC = 0.68 versus 0.52; *P* = 0.008). Using 35 as the most balanced PCA3 cut-off score resulted in sensitivity, specificity, and odds ratio (OR) of 58%, 72%, and 3.6, respectively. Unfortunately, the median PCA3 scores in patients with aggressive PCa (Gleason score <7 versus ≥7) were not significantly different [36]. Nakanishi et al. [37] (*n* = 142) found that PCA3 score was significantly correlated with tumor volume (*P* = 0.008) and an increasing PCA3 score was associated with a higher Gleason score (*P* = 0.005). In a receiver operating curve (ROC) analysis, the PCA3 score could discriminate low volume tumors (<0.5 cc) well with AUC of 0.757. Auprich et al. [38] (*n* = 305) also showed consistently that PCA3 scores were significantly lower in men with low volume tumors and insignificant PCa (*P* < 0.001).

Conversely, Hessels et al. [39] did not find a significant association between PCA3 score in urine sediment after DRE with any PCa prognostic parameter, including Gleason score, tumor volume, or stage. Similarly, Liss et al. [40] reported that PCA3 score did not correlate with adverse pathological features, including stage, Gleason score, or extraprostatic extension.

Based on the promising findings from several studies, urinary PCA3 was further evaluated for its ability to predict biopsy progression in men undergoing AS. Ploussard et al. [41] retrospectively evaluated the performance of PCA3 in men who met criteria for AS, but underwent immediate RP (*n* = 106). A high PCA3 score (≥25) was an important predictive factor for tumor volume ≥ 0.5 cm^3^ and significant cancer, defined as nonorgan confined, or any Gleason pattern 4 or Gleason pattern 5, or tumor volume of at least 0.5 cm^3^, in a multivariate analysis (OR, 5.4; *P* = 0.01 and OR, 12.7; *P* = 0.003, resp.). However, no relationship was observed between PCA3 score and disease stage (*P* = 0.155).

In the first evaluation of urine PCA3 in AS patients enrolled in the Johns Hopkins AS program (*n* = 294) [42], the PCA3 score was not significantly associated with biopsy reclassification (*P* = 0.131), or biopsy Gleason score ≥ 7 (*P* = 0.304), with minimal ability to discriminate unfavorable biopsy pathology (AUC = 0.589; *P* = 0.076). However, in the recently conducted multi-institutional Canary Prostate AS Study (*n* = 387) [43], PCA3 score was significantly associated with a higher biopsy Gleason score and tumor volume, assessed by the percentage of positive cores, in subsequent biopsies (*P* < 0.01 for all comparisons). Using log-transformed biomarker scores as continuous predictors, the OR for a Gleason score of ≥ 7 versus < 7 for PCA3 was 1.67 (95% confidence interval (CI): 1.10–2.52; *P* = 0.02).

In many studies, although PCA3 has clinical utility for detecting PCa, its contribution to prognostic prediction is still contentious. With respect to AS, prognostic value for predicting an unfavorable biopsy conversion at annual surveillance biopsy in the AS program could not be defined due to sparse data. Thus, its role in risk assessment during AS needs to be tested in larger studies with repeated PCA3 score measures.

## 5. TMPRSS2:ERG

TMPRSS2:ERG fusion is a rearrangement of the TMPRSS2 gene, an androgen-regulated transcriptional promoter, and the ERG oncogene, occurring in approximately half of Caucasian patients with PCa [44]. Similar to PCA3, a TMPRSS2:ERG rearrangement can be detected in urine after DRE [45] and can also be normalized to the amount of PSA mRNA to generate a TMPRSS2:ERG score. Hessels et al. [45] reported that detecting TMPRSS2:ERG fusion in urine has high specificity of 93% and 94% positive predictive value (PPV) for PCa detection. Moreover, a population-based study found that TMPRSS2:ERG gene fusion is associated with an increased cumulative incidence ratio of 2.7 for developing metastases and PCa-specific mortality [46].

Tomlins et al. [47] developed a clinical grade, quantitative TMPRSS2:ERG urine assay and measured TMPRSS2:ERG transcript levels in a large-scale multicenter study including a community biopsy cohort (*n* = 471), an academic biopsy cohort (*n* = 623), and prostatectomy cases (*n* = 218). TMPRSS2:ERG score was positively associated with direct markers of tumor volume, including number of positive cores and maximum percentage of positive cores, in both the academic biopsy cohort and the community biopsy cohort. TMPRSS2:ERG score was also significantly higher in men with high prostatectomy Gleason score (>6 versus 6) (*P* = 0.009) and was significantly associated with Gleason score upgrading and significant cancer (*P* = 0.008 and *P* = 0.004, resp.).

In the most recently published prospective multicenter study (*n* = 443) [48], both PCA3 and TMPRSS2:ERG had independent additional predictive value over the European Randomised Study of Screening for Prostate Cancer risk calculator (ERSPC-RC) parameters for predicting PCa in multivariate analyses (OR, 3.64; *P* < 0.001 and OR, 3.28; *P* = 0.002, resp.). The AUC increased incrementally from 0.799 (ERSPC-RC) to 0.833 (ERSPC-RC plus PCA3) to 0.842 (ERSPC plus PCA3 plus TMPRSS2:ERG) to predict PCa. Interestingly, in multivariate logistic regression analyses, only TMPRSS2:ERG added significant predictive value to the ERSPC-RC to predict biopsy Gleason score (OR, 7.16; *P* < 0.001) and clinical tumor stage (OR, 2.60; *P* = 0.023), whereas PCA3 did not.

In AS setting, within the above-mentioned multi-institutional Canary Prostate AS Study (*n* = 387) [43], TMPRSS2:ERG score was also significantly associated with higher biopsy Gleason score and tumor volume, assessed by the percentage of positive cores, in subsequent biopsies (*P* < 0.01 for all comparisons). Using log-transformed biomarker scores as continuous predictors, the OR for a Gleason score of ≥ 7 versus < 7 for TMPRSS2:ERG was 1.24 (95% CI, 1.01–1.53; *P* = 0.05). In a ROC curve analysis, the AUC for predicting a Gleason score ≥ 7 was 0.68 for PSA alone and 0.70 for the combination of both markers (PCA3 and TMPRSS2:ERG) and PSA, respectively. From their results, they showed that both markers are potential predictors to stratify the risk of having aggressive cancer for men on AS.

Whelan et al. [49] investigated secretion capacity biomarkers, including total RNA (TXNRD1 mRNA, PSA mRNA, TMPRSS2:ERG fusion mRNA, and PCA3 mRNA) and specimen volume in expressed prostatic secretion (EPS) specimens before RP from patients who were eligible for AS based on National Comprehensive Cancer Network (NCCN) guidelines (*n* = 216). Two high-performing models were identified, one featuring type III and IV TMPRSS2:ERG variants and another featuring two secretion capacity biomarkers. The AUCs of the TMPRSS2:ERG model and the secretion capacity model for detecting upstaging in the NCCN AS group were 0.80 and 0.79, respectively. Furthermore, the best performing model was associated with a reduced risk of upstaging and of both upstaging and Gleason upgrading by 7.8-fold and 5.2-fold, respectively. Interestingly, these results were supported by Berg et al. [50], who showed a significant association between ERG positivity at diagnosis and the risk of progression during AS (Cox hazard ratio (HR), 2.45; 95% CI, 1.62–3.72; *P* < 0.0001).

## 6. Oncotype DX Prostate Cancer Assay

The Oncotype DX Prostate Cancer Assay is a multigene RT-PCR expression assay that measures expression of 12 cancer-related genes representing four biological pathways and five reference genes in tumor tissue from formalin-fixed paraffin-embedded prostate needle biopsies. Gene expression is normalized by subtracting the aggregated expression of the reference genes and algorithmically combined to calculate the genomic prostate score (GPS) [51]. Some of the key challenges in developing this biopsy-based assay for PCa include the heterogeneous and multifocal nature of the disease and the very small amounts of tumor tissue available from diagnostic prostate needle biopsies.

In a clinical validation study presented at American Urological Association (AUA) annual meeting in 2013 [52], it was reported that GPS, assessed in diagnostic biopsy tissue, strongly predicted high-grade and/or pT3 disease after adjusting for Cancer of the Prostate Risk Assessment (CAPRA) score or other standard pretreatment factors in patients suitable for AS. In the most recently published validation study by Klein et al. (*n* = 395) [53], the biopsy-based 17-gene GPS improved the prediction of the presence or absence of adverse pathology, which may help men diagnosed with PCa decide between AS and immediate definitive treatment. In their study, GPS was strongly associated with clinical recurrence in the RP group (*n* = 441) (HR, 2.32; 95% CI, 1.81–3.00; *P* < 0.001). GPS predicted high-grade and high-stage disease in RP specimens (OR per 20 GPS units, 2.3; 95% CI, 1.5–3.7; *P* < 0.001 and 1.9; 95% CI, 1.3–3.0; *P* = 0.003, resp.) and high-grade and/or high-stage disease after adjusting for CAPRA score with OR of 2.1 (95% CI, 1.4–3.2; *P* < 0.005). Moreover, adding the GPS to the CAPRA score improved the AUC for favorable pathology to 0.67 from 0.63 with the CAPRA score alone. However, this improvement of AUC, as well as the decision-curve analysis, did not show a really perceptible benefit for clinical practice when adding the GPS to other clinical parameters. Moreover, they did not describe detailed information on the biopsy scheme used in the study.

Although GPS could provide additional prognostic information over the existing clinical risk-stratification tools, further validation studies are needed to provide robust evidence.

## 7. Other Potential Biomarkers

Several recent European studies [54–57] have indicated that a panel of four kallikrein markers, including tPSA, fPSA, intact PSA, and kallikrein-related peptidase 2 (hK2), can be used to improve the predictive accuracy of biopsy outcome and reduce unnecessary biopsies. Using data from the Sweden section of the ERSPC (*n* = 740), Vickers et al. [54] reported that a panel of four kallikrein markers showed significantly better predictive accuracy of biopsy outcome in previously unscreened men with elevated PSA compared with PSA alone (AUC from 0.68 to 0.83, *P* < 0.0005, and from 0.72 to 0.84, *P* < 0.0005, without DRE and with DRE, resp.). They estimated that using a 20% risk of prostate cancer as the threshold for biopsy would have reduced the number of biopsies by 424 (57%), while missing only a small number of cancers (31 of 152 low-grade cancers and three of 40 high-grade cancers). Furthermore, in men with a previous negative biopsy but persistently elevated PSA (*n* = 925), Gupta et al. [57] evaluated the performance characteristics of a panel of four kallikrein markers to determine the predictive value of repeat biopsy outcome in the Rotterdam section of the ERSPC. The full-kallikrein panel, incorporating age and DRE, had higher discriminative accuracy than PSA and DRE alone for predicting high-grade cancer (Gleason score ≥7) at biopsy with the AUC improving from 0.76 to 0.87 (*P* = 0.003). Additionally, these markers had an improved ability to distinguish between pathologically insignificant and aggressive disease on pathologic examination of RP specimens in a recently published study from the Rotterdam section of the ERSPC (*n* = 392) [58]. Although the clinical model had good accuracy for predicting aggressive disease (AUC, 0.81), the panel of four kallikrein markers enhanced the base model, with an AUC of 0.84 (*P* < 0.0005). Moreover, this improvement was more remarkable in low- and very low-risk patients (AUC from 0.75 to 0.81 and from 0.72 to 0.81, resp.). Clinical application of the model incorporating these kallikrein markers would reduce rates of surgery by 135 of 1000 patients overall and 110 of 334 patients with pathologically insignificant disease [58].

The panel of four kallikrein markers was combined to generate the 4K score. The 4K score test was developed by OPKO Lab, a division of OPKO Health, and is being commercialized. Thus, it may soon be available for clinical settings. However, so far no evidence for the usefulness of the four kallikrein panel in AS programs has been presented.

The expression levels of different cell cycle progression (CCP) genes are highly correlated with cell proliferation, presumably reflecting the fraction of actively dividing cells within the sampled tissue [59]. The expression levels of these genes were known to be significantly associated with the risk of disease progression [60–62]. The CCP score (Prolaris) is calculated as the average expression level of 31 CCP genes, normalized to 15 housekeeper genes [60], which was developed as a clinical laboratory test by Myriad Genetics, Inc. The expression levels of these genes were measured using quantitative RT-PCR on RNA from formalin-fixed paraffin-embedded tumor samples.

Cuzick et al. [60] showed that CCP score provides a substantial amount of independent information regarding the risk of recurrence after RP (HR for a 1-unit increase in CCP score, 1.74; 95% CI, 1.39–2.17; *P* = 3.3 × 10^−6^) and the risk of death in conservatively managed PCa diagnosed by transurethral resection of the prostate (TURP) (HR, 2.57; 95% CI, 1.93–3.43; *P* = 8.2 × 10^−11^) in large retrospective cohorts (*n* = 366 and *n* = 337, resp.). The same group reported on the ability of the CCP score to predict death from PCa in a cohort of men with clinically localized disease diagnosed by a needle biopsy and managed conservatively (*n* = 349) [61]. In their subsequent study, the CCP score was the strongest independent predictor of cancer death outcome for conservatively managed patients (HR, 2.02; 95% CI, 1.62–2.53; *P* < 10^−9^).

The CCP score also had significant prognostic accuracy in an academic RP cohort study for validation (*n* = 413) [62] after controlling for all available clinical and pathologic data. With or without adjusting for clinical variables, increasing CCP score was associated with markedly higher hazards for biochemical progression (HR, 2.1; 95% CI, 1.6–2.9; *P* < 0.001 in univariate analysis and HR, 1.7; 95% CI, 1.3–2.4; *P* < 0.001 with adjustment for CAPRA score, resp.). Moreover, the combined CAPRA and CCP score improved the concordance index for both the overall cohort and low-risk patients (0.77 versus 0.73 for CAPRA score alone).

Additional validation studies are under way using biopsy specimens from pre-RP and AS cohorts, which will help define the role of the CCP score in AS setting. However, there is still no definite evidence that histopathologic markers have clinical utility for patient selection and monitoring during AS.

## 8. Discussion

We have provided insight into the value of novel biomarkers that could be used for patient selection and follow-up on AS for low-risk PCa.  Table 2 shows a summary of studies investigating the prognostic value of novel biomarkers in AS. Several of these novel biomarkers have the potential to improve the current practice of AS. In many series, MP-MRI showed promising results because of the very high negative predictive value (NPV) for significant PCa [63–66]. Thus, if validated, favorable MRI findings on a good-quality MP-MRI may be used for selection and follow-up of patients during AS and might obviate the need for repeat biopsies. In addition to promising imaging tools, several serum, urinary, and tissue biomarkers have been intensively investigated to determine their additional value for cancer detection and prognosis. However, a biomarker must demonstrate evidence of strong analytical validity, clinical validity, and clinical utility to enter wide clinical practice.

Many studies have shown that %[−2]proPSA and PHI are more accurate than the currently used PSA and other PSA derivatives for predicting the presence of PCa and aggressiveness, which might result in the avoidance of unnecessary biopsies without missing significant PCa. The results reported above show that %[−2]proPSA and PHI are particularly useful in patients with the PSA gray zone range of between 2.0 and 10.0 ng/mL, which might lead to reducing unnecessary biopsies in AS patients. Although studies evaluating their potential role in AS program are currently scarce, most results to date are promising. However, additional validation is warranted to determine whether clinically useful thresholds can be defined and to better characterize their role in conjunction with other biomarkers during monitoring patients in AS.

Although PCA3 has been reported to have clinical utility for detecting PCa in many studies, its contribution to prognostic prediction remains controversial. The consensus in most studies is that PCA3 is often correlated with insignificant PCa and tumor volume, yet, in the clinically significant cancers, there is no definite evidence for an association with histopathologic prognostic factors. Considering the heterogeneous character of the disease, combining PCA3 with other biomarkers might be a better option to improve diagnostic and prognostic accuracy instead of using a single prognostic variable.

TMPRSS2:ERG is highly specific for predicting clinically significant PCa on biopsy, despite the relatively low sensitivity. Robert et al. provided a rational basis for combining PCA3 and TMPRSS2:ERG in tissue samples [67]. After the first study on combining PCA3 and TMPRSS2:ERG reported by Hessels et al. [45], several studies [48, 68–70] showed better accuracy of the combination with TMPRSS2:ERG than PCA3 alone for the prediction of PCa detection and progression. These encouraging results for combined biomarkers may help to improve the prediction of biopsy reclassification during AS and pathologic features at RP. However, additional multi-institutional studies on larger populations are needed to verify if these combined biomarkers improve the prediction of biopsy reclassification in AS patients.

The panel of four kallikrein markers also has good predictive accuracy for biopsy outcome and aggressive disease, and tissue biomarkers (i.e., Oncotype DX Prostate Cancer Assay, CCP score) show promising ability for predicting disease progression. There is a growing recognition that molecular biomarkers can complement conventional clinical and pathologic parameters to personalize the care of patients with cancer. However, incorporating these biomarkers into standard clinical practice requires a level of validation that is not often achieved. Unquestionably, these markers also need to be verified with a higher level of evidence for clinical validation and usefulness in AS programs in the future.

Nevertheless, the present systematic review of the literature on novel biomarkers has several limitations. First, most of the studies were retrospective, and different biopsy protocols were used, possibly causing significant heterogeneity. Further heterogeneity was found regarding study design (i.e., retrospective versus prospective, recruitment strategy) and population characteristics (i.e., age, race, and total PSA range). Second, the definition of clinical significance and disease progression was arbitrary. Third, most studies were limited to intermediate endpoints such as biopsy reclassification, treatment-free survival, or pathologic findings in RP specimens. No data are available with respect to longer term endpoints such as time to metastasis or prostate cancer-specific mortality.

The majority of biomarkers published during the last few years are still in the investigation or validation phase. Although several of these novel biomarkers showed improved predictive accuracy than that of classical parameters, there is still a need of a standard study design to avoid common bias and clinical validation. Further studies are required to define how these novel biomarkers could be used to select men that would most benefit from an AS program and how these markers could be incorporated into the follow-up schedule in AS patients.

## 9. Conclusions

Several biomarkers, which could be novel tools to improve PCa risk assessment, showed promising prognostic value. %[−2]proPSA and PHI showed improved predictive value for an unfavorable biopsy conversion at annual surveillance biopsy in the AS program. PCA3 and TMPRSS2:ERG had additional independent predictive value for the prediction of PCa detection and progression, although PCA3 was limited in predicting aggressive cancer. Nevertheless, both biomarkers improved the multivariate accuracy for predicting biopsy outcome when combined with each other. Other tissue biomarkers also showed promising ability to predict disease progression.

Implementing these promising novel biomarkers into clinical practice may not only increase the number of patients suitable for AS but also reduce the burden of monitoring during AS. However, there is a great need for further well-designed, large, multicenter, prospective studies, to validate the currently available biomarkers and identify an optimal combination of biomarkers and optimal thresholds for each biomarker.

## Figures and Tables

**Table 1:** AUCs for PSA, %fPSA, %[−2]proPSA, and PHI, and relationship between %[−2]proPSA and PHI and Gleason score.

Reference	AUC PSA	AUC %fPSA	AUC %[−2]proPSA	AUC PHI	Relationship of %[−2]proPSA and GS	Relationship of PHI and GS
	(95% CI)	(95% CI)	(95% CI)	(95% CI)		
Catalona et al., 2011 [19] (*n* = 892)	0.525	0.648	Not reported	0.703	Not reported	The probability of GS ≥ 7 was 26.1% when PHI < 25 and 42.1% when PHI ≥ 55
Jansen et al., 2010 [20]						
Rotterdam (*n* = 405)	0.585 (0.535–0.634)	0.675 (0.627–0.721)	0.716 (0.669–0.759)	0.750 (0.704–0.791)	%[−2]proPSA discriminates GS ≥ 7 (with biopsy GS, *P* = 0.002; with pathologic GS, *P* = 0.09)	PHI discriminates GS ≥ 7 (with biopsy GS, *P*: <0.0001; with pathologic GS, *P* = 0.02)
Innsbruck (*n* = 351)	0.543 (0.473–0.594)	0.576 (0.523–0.629)	0.695 (0.644–0.743)	0.709 (0.658–0.756)	No (neither with biopsy or with pathologic GS)	No (neither with biopsy nor with pathologic GS)
Sokoll et al., 2010 [21] (*n* = 556)	0.58 (0.53–0.64)	0.66 (0.61–0.71)	0.70 (0.65–0.75)	0.76 (0.72–0.81)	%[−2]proPSA increased with increasing GS (*P* = 0.02)	Not reported
Lazzeri et al., 2013^*^ [22] (*n* = 646)	0.50 (0.46–0.54)	0.64 (0.61–0.68)	0.67 (0.64–0.71)	0.67 (0.64–0.71)	Significant (Spearman *r*: 0.245; *P* < 0.001), and it did improve the prediction of GS ≥ 7 PCa in multivariable accuracy analysis by 7.3%	Significant (Spearman *r*: 0.276; *P* < 0.001), and it did improve the prediction of GS ≥ 7 PCa in multivariable accuracy analysis by 7.6%
Lazzeri et al., 2013^**^ [23] (*n* = 158)	0.55 (0.47–0.63)	0.60 (0.52–0.68)	0.73 (0.66–0.80)	0.73 (0.66–0.80)	Significant (Spearman *r*: 0.366; *P* = 0.002), but it did not improve the prediction of GS ≥ 7 PCa in multivariable accuracy analysis	Significant (Spearman *r*: 0.464; *P* < 0.001), but it did not improve the prediction of GS ≥ 7 PCa in multivariable accuracy analysis
Stephan et al., 2013 [24] (*n* = 1,362)	0.56 (0.53–0.59)	0.61 (0.59–0.64)	0.72 (0.70–0.75)	0.74 (0.71–0.76)	Significantly higher median values of %[−2]proPSA were observed for patients with GS ≥ 7 (%[−2]proPSA = 2.68) compared with a GS < 7 (%[−2]proPSA = 2.34; *P* = 0.011)	Significantly higher median values of phi were observed for patients with GS ≥ 7 (PHI = 59.7) compared with a GS < 7 (PHI = 53.1; *P* = 0.002)
Ng et al., 2014 [25] (*n* = 230)	0.547 (0.421–0.674)	0.572 (0.437–0.708)	0.768 (0.660–0.876)	0.781 (0.675–0.887)	Not reported	Not reported

AUC: area under the curve; PCa: prostate cancer; PSA: prostate-specific antigen; %fPSA: percentage of free PSA to total PSA; %[−2]proPSA: percentage of [−2]proPSA to free PSA; PHI: Prostate Health Index; GS: Gleason score; CI: confidence interval.

^*^An observational, prospective, multicenter European cohort, the PROMEtheuS project.

^**^A nested case-control study from the same PROMEtheuS database.

**Table 2:** Studies investigating the prognostic value of novel biomarkers in active surveillance.

Biomarkers	Reference	Year	*n*	Predictor variables	Study endpoint(s)	Results
%[−2]proPSA, PHI	Makarov et al. [27]	2009	71	[−2]proPSA/%fPSA	Biopsy progression	[−2]proPSA/%fPSA was significantly associated with unfavorable biopsy in repeat biopsy (Cox HR, 2.53; 95% CI, 1.18–5.41; *P* = 0.02).
	Isharwal et al. [28]	2011	71	[−2]proPSA/%fPSA, PHI, and biopsy tissue DNA content	Biopsy progression	PHI and [−2]proPSA/%fPSA showed improvement in the predictive accuracy (c-index, 0.6908 and 0.6884, resp.) for unfavorable biopsy conversion in the multivariate models including the biopsy tissue DNA content.
	Tosoian et al. [29]	2012	167	%fPSA, %[−2]proPSA, [−2]proPSA/%fPSA, and PHI	Biopsy progression	Baseline and longitudinal measurements of %fPSA, %[−2]proPSA, [−2]proPSA/%fPSA, and PHI demonstrated significant associations with biopsy reclassification, and %[−2]proPSA and PHI provided the greatest predictive accuracy for high-grade cancer.
	Hirama et al. [30]	2014	134	%[−2]proPSA, PHI	Biopsy progression	Baseline %[−2]proPSA and PHI were the only independent predictive factors for pathological upgrading in multivariate logistic regression analysis (*P* = 0.008 and *P* = 0.008, resp.).

PCA3	Ploussard et al. [41]	2011	106	PCA3 score	Prognostic pathologic findings in RP specimens	The risk of having a cancer ≥0.5 cm^3^ and a significant PCa was increased by 3-fold in men with a PCA3 score of ≥25 compared with men with a PCA score of <25. In a multivariate analysis, a high PCA3 score (≥25) was an important predictive factor for tumor volume ≥0.5 cm^3^ (OR: 5.4; *P* = 0.010) and significant PCa (OR: 12.7; *P* = 0.003).
	Tosoian et al. [42]	2010	294	PCA3 score	Biopsy progression	PCA3 alone could not be used to identify men with progression on biopsy (AUC, 0.589; 95% CI, 0.496–0.683; *P* = 0.076). After adjustment for age and date of diagnosis, PCA3 was not significantly associated with progression on biopsy (*P* = 0.15).
	Lin et al. [43]	2013	387	PCA3 score	Biopsy progression	PCA3 score was significantly associated with a higher biopsy Gleason score and tumor volume in subsequent biopsies (*P* < 0.01 for all comparisons). Using log-transformed biomarker scores as continuous predictors, the OR for a Gleason score of ≥7 versus <7 for PCA3 was 1.67 (95% CI: 1.10–2.52; *P* = 0.02)

TMPRSS2:ERG	Lin et al. [43]	2013	387	TMPRSS2:ERG	Biopsy progression	TMPRSS2:ERG score was significantly associated with a higher biopsy Gleason score and tumor volume in subsequent biopsies (*P* < 0.01 for all comparisons). Using log-transformed biomarker scores as continuous predictors, the OR for a Gleason score of ≥7 versus <7 for TMPRSS2:ERG was 1.24 (95% CI: 1.01–1.53; *P* = 0.05).
	Whelan et al. [49]	2013	216	TMPRSS2:ERG model Secretion capacity model (PSA, total EPS RNA, and total EPS volume)	Upgrading Upstaging in RP specimens	The AUCs of the TMPRSS2:ERG model and the secretion capacity models for detecting upstaging in the NCCN AS group were 0.80 and 0.79, respectively. TMPRSS2:ERG model was associated with a reduced risk of upstaging and of both upstaging and Gleason upgrading by 2.4-fold and 2.7-fold, respectively (*P* = 0.1041 and *P* = 0.1576, resp.).
	Berg et al. [50]	2014	265	ERG positivity	Clinical progression Biopsy progression PSA progression	The ERG-positive group showed significantly higher incidences of overall AS progression (*P* < 0.0001) and of the subgroups PSA progression (*P* < 0.0001) and biopsy progression (*P* < 0.0001). ERG positivity was a significant predictor of overall AS progression in multiple Cox regression (HR, 2.45; 95% CI, 1.62–3.72; *P* < 0.0001).

%[−2]proPSA: percentage of [−2]proPSA to free PSA; PHI: Prostate Health Index; %fPSA: percentage of free PSA to total PSA; HR: hazard ratio; CI: confidence interval; RP: radical prostatectomy; PCa: prostate cancer; OR: odds ratio; AUC: area under the curve; PSA: prostate-specific antigen; EPS: expressed prostatic secretion; NCCN: National Comprehensive Cancer Network; AS: active surveillance.
